# Influence of age and co-medication on dolutegravir glucuronidation in paediatric patients

**DOI:** 10.1111/bcp.16238

**Published:** 2024-09-03

**Authors:** Tom G. Jacobs, Hylke Waalewijn, Lily Houlden, Pauline D.J. Bollen, Annet Nanduudu, Esether Nambi, Haseena Cassim, Abbas Lugemwa, Shafic Makumbi, Lara N. Monkiewicz, Clare Shakeshaft, Alasdair Bamford, Moherndran Archary, Godfrey Musuro, Ennie Chidziva, Hilda A. Mujuru, Mutsa Bwakura-Dangarembizi, Chishala Chabala, Anna Turkova, Di M. Gibb, Mark F. Cotton, Rob Aarnoutse, David M. Burger, Angela Colbers

**Affiliations:** 1 Department of Pharmacy, Research Institute for Medical Innovation, Radboud University Medical Center, Nijmegen, The Netherlands; 2 Division of Clinical Pharmacology, Department of Medicine, University of Cape Town, Cape Town, South Africa; 3 Medical Research Council Clinical Trials Unit, University College London, London, UK; 4 Hospital Gelderse Vallei, Ede, The Netherlands; 5 Joint Clinical Research Centre, Kampala, Uganda; 6 Perinatal HIV Research Unit, University of the Witwatersrand, Johannesburg, South Africa; 7 Joint Clinical Research Centre, Mbarara, Uganda; 8 Durban International Clinical Research Site, Durban, South Africa; 9 University of Zimbabwe Clinical Research Centre, Harare, Zimbabwe; 10 Department of Paediatrics and Child Health, School of Medicine, University of Zambia, Lusaka, Zambia; 11 Children’s Hospital, University Teaching Hospitals, Lusaka, Zambia; 12 Family Centre for Research with Ubuntu, Stellenbosch University, Cape Town, South Africa

**Keywords:** children, dolutegravir, drug−drug interaction, glucuronidation, HIV, pharmacokinetics, UGT1A1

## Abstract

Dolutegravir (DTG) is primarily metabolized by uridine diphosphate glucuronosyl-transferases, forming the pharmacologically inactive DTG glucuronide (DTG-gluc). We described the dolutegravir metabolic ratio (DTG-MR; DTG-gluc AUC_0−24h_ divided by DTG AUC_0−24h_) in 85 children with HIV aged 3 months to 18 years receiving DTG in the CHAPAS-4 (ISRCTN22964075) and ODYSSEY (NCT02259127) trials. Additionally, we assessed the influence of age, body weight, nucleoside/nucleotide reverse transcriptase inhibitor (NRTI) backbone, rifampicin use and kidney function on DTG-MR. The overall geometric mean (CV%) DTG-MR was 0.054 (52%). Rifampicin use was the only significant factor associated with DTG-MR (*P* <.001) in multiple linear regression. DTG-MR geometric mean ratio was 1.81 (95% CI: 1.57–2.08) for children while on *vs*. off rifampicin. This study showed that overall DTG-MR in children was similar to adults, unaffected by age or NRTI backbone, and increased with rifampicin co-administration. These findings support future paediatric pharmacokinetic modelling and extrapolation from adult data.

## 1 Introduction

An estimated 1.5 million children below 15 years old were living with HIV globally in 2022.^1^ Recently, dolutegravir (DTG)-based antiretroviral therapy (ART) was found to be superior to prior standard of care for children living with HIV.^2^ Therefore, DTG became preferred treatment in international paediatric ART guidelines with rapid global roll-out of adult and paediatric DTG drug formulations.^3,4^

Multiple studies have confirmed adequacy of paediatric DTG dosing using WHO-recommended weight-band dosing.^5–7^ These studies also reported more variable DTG exposure in plasma for children than adults based on mg/kg dose, highlighting the need for further investigation of potential mechanisms for this high variation.^5–7^ DTG is predominantly metabolized by uridine diphosphate-glucuronosyl transferase (UGT) and to a lesser extent by cytochrome p450 3A (CYP3A) enzymes. Its main inactive metabolite, DTG-glucuronide (DTG-gluc), is formed by conjugation via UGT1A1 (major), UGT1A3 (minor) and UGT1A9 (minor).^8^ Total plasma exposure to DTG-gluc divided by total DTG exposure is a measure for the DTG glucuronidation metabolic plasma ratio (DTG-MR).^8^ DTG-MR is a phenotypic parameter for DTG metabolism through UGTs and is therefore an important measure for the clearance of DTG.

At birth, UGT1A1 activity is very low (10%–30% of adult levels), reaching adult levels after about 3–6 months.^9^ Interindividual variability of UGT activity is high in young children, which might contribute to more variable DTG exposure in children than adults.^5–7^

Furthermore, DTG plasma concentrations can be affected by drug–drug interactions. Rifampicin, a commonly used drug to treat tuberculosis, is a strong inducer of CYP3A4 and to a lesser extent of UGT1A1, thereby increasing metabolism of DTG.^10–12^ To overcome this interaction, DTG is currently recommended to be administered twice daily instead of once daily when co-administered with rifampicin.^10,13^ Additionally, a recent study reported a trend towards lower DTG concentrations in children taking a tenofovir alafenamide (TAF)-containing backbone compared to other nucleoside/ nucleotide reverse transcriptase inhibitor (NRTI) backbones.^14^ However, this study was not designed as a formal pharmacokinetic interaction study. The DTG-MR could provide mechanistic understanding of these findings.

Studying the DTG-MR in children could contribute to a better mechanistic understanding of how DTG metabolism is affected by age and co-medication and could inform future paediatric pharmacokinetic modelling studies. Therefore, the aim of this study was (1) to describe the DTG-MR in a paediatric population and (2) to identify sources of variability in DTG-MR and to specifically assess the effect of age and rifampicin.

## 2 Methods

### 2.1 Procedures and participants

Plasma samples from pharmacokinetic sub-studies of the CHAPAS-4 (ISRCTN22964075) and ODYSSEY (NCT02259127) trials were used to measure DTG and DTG-gluc concentrations. Children were enrolled at various hospitals in South Africa, Uganda and Zimbabwe, all receiving DTG dispersible tablets (DT) or film-coated tablets (FCT) following WHO weight-band dosing.^4^ We included all children enrolled in the CHAPAS-4 pharmacokinetic sub-studies receiving DTG.^14^ Additionally, a subset of children was selected from pharmacokinetic sub-studies within the ODYSSEY trial; all children aged <2 years, a random sample of older children using the randomisation tool in Microsoft® Excel,^5,6^ also all children enrolled in the tuberculosis (TB-PK) sub-study.^13^ We included samples taken while on concomitant rifampicin as well as without rifampicin treatment (4 weeks after completion of the TB treatment) for the TB-PK sub-study.^13^ Exclusion criteria included missing plasma samples in the main trial, or no left-over plasma, resulting in insufficient DTG concentration datapoints to calculate DTG AUC and subsequently DTG-MR, and using medications, other than rifampicin, known to interact with DTG.

### 2.2 Bioanalysis

Concentrations of plasma DTG and DTG-gluc were determined using a validated ultraperformance liquid chromatography–tandem mass spectrometry (UPLC-MS/MS) quantification method, described by Bollen et al.^15^ The DTG assay was externally validated through an international interlaboratory quality control programme.^16^

### 2.3 Metabolic ratio

DTG AUC_0−24h,_ DTG-gluc AUC_0−24h_ and DTG apparent clearance were assessed by non-compartmental analysis using the trapezoidal method (linear up, log down), and summary statistics were calculated using Phoenix WinNonlin 64 (version 8.1; Certara, Princeton, NJ, USA). The DTG-MR was determined by dividing the 24-h area under curve (AUC_0−24h_) of DTG-gluc by the AUC_0−24h_ of DTG, corrected for molar mass. DTG and DTG-gluc concentrations were available for CHAPAS-4 participants for timepoints *t* = 0 (pre-dose), 1, 2, 4, 6, 8, 12 and 24 h after drug administration, while, for ODYSSEY participants, *t* = 2, 6 and 24 h timepoints were used to calculate DTG and DTG-gluc AUC_0−24h_. DTG and DTG-gluc *t* = 0 h concentrations were equalled to *t* = 24 h concentrations to facilitate calculation of AUC_0−24h_ for ODYSSEY data.

### 2.4 Statistical analysis

All statistical analyses were performed using IBM SPSS Statistics for Windows (v29.0) with a significance level of 0.05.

To describe the DTG-MR in the population in terms of central tendency and spread, we reported the overall geometric mean with the coefficient of variation and range (min–max) for all children. To identify sources of variability in DTG-MR, we then assessed the effect of age, body weight, type of NRTI backbone, concomitant rifampicin use and kidney function on the DTG-MR in children using multiple linear regression. Only one DTG-MR per child was included in the multiple linear regression analysis. For children in the TB-PK study with DTG-MR measurements on and off rifampicin, the value while on rifampicin was used for the multiple linear regression. Kidney function was defined as estimated glomerular filtration rate (eGFR; bedside Schwartz formula^17^) and was calculated using serum creatinine values obtained at a regular study visit close to the pharmacokinetic sub-study visit.

Finally, a more in-depth analysis on the possible effect of age and concomitant use of rifampicin on the DTG-MR was done. We applied Pearson’s correlation test to assess the association between age and log-transformed DTG-MR for children ≤6 months old as maturation of UGT1A1 may still be ongoing for these infants. The effect of concomitant rifampicin on DTG-MR was defined as geometric mean ratio (DTG-MR with rifampicin/DTG-MR without rifampicin) with 95% confidence interval, which is equivalent to a paired t-test on log-transformed DTG-MR values. All children included in ODYSSEY TB-PK with a DTG-MR while on rifampicin and off rifampicin were included in this analysis.

### 2.5 Ethics

The main CHAPAS-4 (University College London Research Ethics Committee, number: 11205/001) and ODYSSEY (National Research Ethics Service Committee London–Riverside, number: 15/LO/1120) trials and sub-studies were approved by local and national ethics committees. All caretakers provided written consent for participation of the children in the studies and to use the saved samples for further analyses. In addition, older children provided written assent as per local guidelines.

## 3 Results

Ninety Black African children (age range: 3 months to 18 years) were considered in this study; 49 children from the ODYSSEY PK sub-studies (60 DTG-MR measurements) and 41 children from the CHAPAS-4 trial (41 DTG-MR measurements). All samples had detectable DTG and DTG-gluc concentrations. Five children were excluded, four of whom had either missing DTG-gluc concentration data for concentrations below LLOQ or had insufficient residual plasma. The fifth child was on concomitant valproic acid, affecting DTG pharmaco-kinetics. The characteristics of all participants are shown in Table 1.

**Figure 1:**
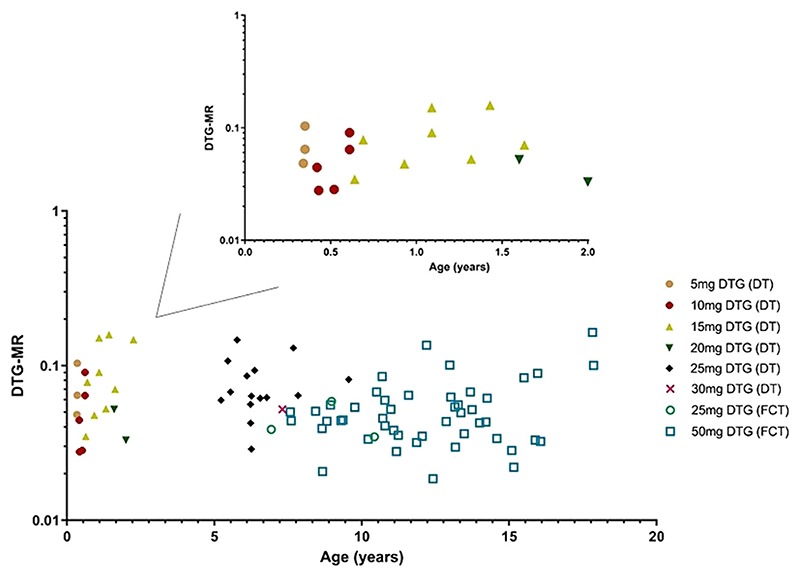
Dolutegravir metabolic glucuronidation ratio over the full age range (lower graph) and for infants under 2 years old (upper graph). The various doses and corresponding tablet formulation are shown as different symbols. Abbreviations: DT, dispersible tablet; DTG, dolutegravir; DTG-MR, dolutegravir metabolic glucuronidation ratio; FCT, film-coated tablet.

In the cohort of children (*n* = 85) not receiving rifampicin, the overall geometric mean (%CV) of DTG-MR was 0.054 (52%), and for children under 6 months old (*n* = 5), it was 0.052 (52%). The variability of DTG-MR within the population showed a notable ninefold range, from 0.019 to 0.16 (see Figure 1).

The multiple linear regression model showed a significant weak correlation with DTG-MR (*R*^2^ = 0.159, *F*(7,76) = 5.183, *P* =.005). Among the included independent variables, only concomitant rifampicin significantly contributed to the model (*P* <.001).

**Figure 2:**
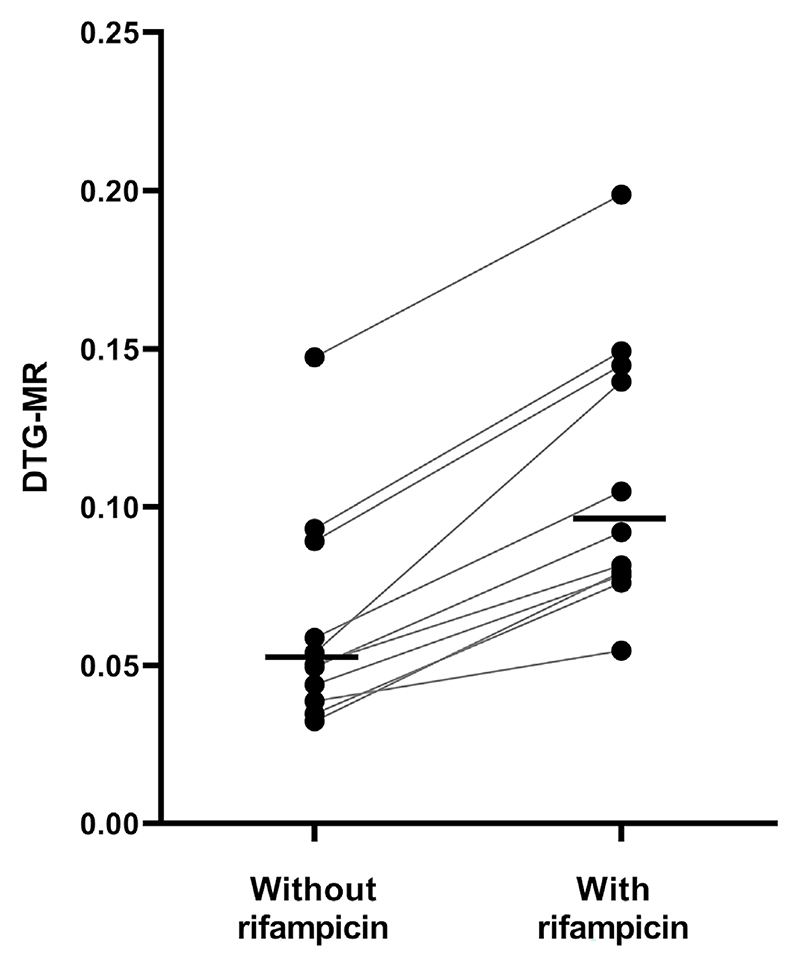
Individual DTG-MR values for children with and without rifampicin. The black horizontal lines represent the geometric mean values. Abbreviations: DTG-MR, dolutegravir glucuronidation metabolic ratio.

In the age subgroup analysis of children aged ≤6 months, no significant correlation was found between age and DTG-MR (*r*(3) = −0.73, *P* =.16). DTG-MR of all included children by age is presented in Figure 1. Regarding the in-depth analysis for concomitant rifampicin treatment, 11 children had an evaluable DTG-MR during co-administration of rifampicin and without rifampicin. The geometric mean (%CV) DTG-MR for those on rifampicin was 0.102 (40%) *vs*. 0.056 (49%) for children without rifampicin, resulting in a geometric mean ratio (with rifampicin/without rifampicin) of 1.81 (95% CI 1.57−2.08) (see Figure 2).

## 4 Discussion and Conclusion

This study is, to our knowledge, the first study to describe the DTG-MR in a large population of children and to identify potential sources of variability of DTG glucuronidation. We found a geometric mean DTG-MR of 0.054 among children aged 3 months to 18 years with a substantial interindividual variation spanning about ninefold. Age, body weight, renal function and the type of NRTI backbone did not affect DTG-MR in our population. However, concomitant rifampicin was associated with a nearly twofold increased DTG-MR.

The geometric mean DTG-MR in the total paediatric population was 0.054, comparable to a previous study in postpartum women living with HIV (median DTG-MR of 0.08, IQR 0.05–0.09) receiving 50 mg DTG daily.^15^ The interindividual variability was large in our population (range: 0.019–0.16). Given that DTG is primarily metabolized via UGT enzymes with minor contributions from other pathways, this variability in DTG-MR likely reflects the large interindividual differences in DTG clearance seen in children compared to adults. Another study showed a higher DTG-MR in HIV-negative people with chronic kidney disease compared to healthy adults (0.055 *vs*. 0.011) after a single 50 mg DTG dose, attributed to reduced renal clearance of DTG-gluc.^18^ Our study found no significant impact of renal function on DTG-MR. However, children with reduced renal function were under-represented in our paediatric population with only four children having an eGFR below 60 mL/min/1.73m^2^. Additionally, no difference in DTG-MR with increasing age was observed. This finding may be explained by the UGT1A1 abundance being similar in children and adults throughout most of childhood, with UGT1A1 maturation occurring within the first 3–6 months of life.^9^ Only five children below 6 months of age were included in this study and none below 3 months old because we could only use data from children enrolled in previous PK studies, which did not include a larger number of young infants. Hence, we were unable to capture the period where most of the enzyme maturation occurs. Another study reported a lower DTG-MR (0.0292) in six infants between 1 and 12 months old.^19^ Furthermore, data from a single neonate suggest very low DTG-MR in neonates, consistent with low activity of UGTs in this population.^20^

UGT1A1 induction by rifampicin was demonstrated by the 1.81-fold increase in DTG-MR seen in children taking concomitant rifampicin. This is consistent with findings from an ex vivo study showing a twofold induction of UGT1A1 in human hepatocytes by rifampicin.^21^ Furthermore, a 2.3-fold higher DTG-MR was reported in infants receiving dolutegravir with rifampicin *vs*. those without rifampicin.^19^ The type of NRTI backbone did not affect the geometric mean DTG-MR. For the probable trend towards lower DTG exposure in children using a TAF-based backbone reported by Bevers et al, we did not find mechanistic evidence based on altered DTG-MR. This is not surprising as TAF is believed not to interfere with drug metabolism through UGT1A1.^22^

Our study had several limitations. Firstly, we were unable to measure metabolites other than the DTG-gluc metabolite. Although DTG-MR represents the main metabolic pathway of DTG, other metabolic enzymes, such as CYP3A4, also play a role in the clearance of DTG. Secondly, we found that renal function did not seem to affect DTG-MR in our population. However, factors, such as variation in activity of hepatic and renal efflux and uptake transporters could contribute to DTG-gluc elimination and, thus, potentially to the DTG-MR.^23^ As data about these factors were unavailable, we could not consider them in the analysis. Thirdly, as genotyping was not done, we could not correct for poor, intermediate or rapid UGT metabolizers, which does impact DTG clearance in children.^24^

In conclusion, our study revealed a considerable interpatient variability in DTG-MR among children aged 3 months to 18 years, yet the mean DTG-MR was comparable to adult values. The DTG-MR was not affected by age, eGFR or NRTI backbone. However, concomitant rifampicin treatment increased DTG-MR, consistent with increased DTG clearance due to induction UGT1A1. These findings can inform future mechanistic dolutegravir pharmacokinetic modelling studies in children and support extrapolating adult pharmacokinetic results for medications undergoing glucuronidation while accounting for the high variability in children. Further studies could explore the role other factors contributing to variation in DTG exposure in children.

## Figures and Tables

**Table 1:** Participant demographics reported as median (IQR), the distribution of children included per sub-analysis, and dolutegravir metabolic ratio per sub-analysis.

Demographics	CHAPAS4 PK	ODYSSEY PK	ODYSSEY TB-PK	All children
N	41	34^a^	11^a^	85^a^
Age (years)	10.8 (7.7−13.4)	1.8 (0.6−10.3)	10.3 (7.0−13.3)	8.98 (5.5−12.9)
Children ≤6 months old	—	5	—	—
Weight (kg)	27.0 (19.6−35.5)	10.5 (6.7−28.1)	27.9 (20.1−32.1)	23.5 (15.9−31.2)
eGFR (mL/min/1.73m^2^)	112 (97−129)	108 (73−129)	100 (84−123)	110 (91−128)
NRTI backbone				
ABC/3TC	12	33	10	54
TAF/3TC	21			21
TDF/3TC			1	1
ZDV/3TC	8	1		9
DTG-MR^b^	0.051 (47%; 0.020− 0.15)	0.59 (53%; 0.028−0.16)	0.102 (40%; 0.055−0.20)/0.056 (49%; 0.019− 0.15)^c^	0.054 (52%; 0.019− 0.16)

Abbreviations: 3TC, lamivudine; ABC, abacavir; DTG-MR, DTG glucuronidation metabolic ratio; NRTI, nucleoside/nucleotide reverse transcriptase inhibitor; TAF, tenofovir alafenamide; TDF, tenofovir disoproxil fumarate; ZDV, zidovudine.

a One subject was included in both ODYSSEY-PK and TB-PK and hence counted as 1 child in the ‘all children’ column.

b DTG-MR reported as geometric mean (%geometric mean coefficient of variation; range).

c DTG-MR while on and off rifampicin, respectively.

## Data Availability

The data supporting the results and analyses presented in this paper are available upon reasonable request from the corresponding author.
